# Mitochondrial Markers for the Detection of Duck Breeds Using Polymerase Chain Reaction

**DOI:** 10.3390/genes12060857

**Published:** 2021-06-03

**Authors:** Małgorzata Natonek-Wiśniewska, Piotr Krzyścin, Dominika Rubiś

**Affiliations:** Department of Animal Molecular Biology, National Research Institute of Animal Production, Krakowska 1, 32-083 Balice, Poland; piotr.krzyscin@iz.edu.pl (P.K.); dominika.rubis@iz.edu.pl (D.R.)

**Keywords:** 12S rRNA, 16S rRNA, Rouen, MH078252, identification of duck DNA, mtDNA of duck

## Abstract

Species identification of the components of various duck breeds has revealed that the lowest identifiable number of components depends on the breed. The results (shown on the agarose gel) of a species-specific PCR reaction for Rouen ducks were less intense than the results for the same amount of components from other popular duck breeds, suggesting differences in the Rouen duck genome. Therefore, the present study aimed to identify part of the Rouen duck’s gene sequences and to develop two new primer pairs. The first pair enables breed-independent identification of duck DNA, and the second distinguishes Rouen ducks from Chinese and Indian Runner ducks. The sequencing reaction yielded sequences of 1386 bp in length, and the identified sequence differs by around 7% from the sequences of Chinese duck species. The detected sequence contributes to improving species identification methods for duck DNA. On its basis, two primers for the identification of duck DNA were designed. The first allows for DNA amplification with the same sensitivity regardless of duck breed. The second primer’s pair is breed specific, and it distinguishes Rouen ducks from Chinese and Indian Runner ducks. Both methods are very sensitive (0.05%).

## 1. Introduction

Conserved sequences in mitochondrial DNA such as 12S rRNA, 16S rRNA, and cytB are widely used seven in species identification methods [1,2,3,4,5,6]. Mitochondrial genome sequences are uniform within a species, but they vary among closely genetically related species. These differences often concern several nucleotides and allow for discrimination between species [4].

However, within-species homogeneity can sometimes be slightly disrupted. This disruption has been observed when developing a method for species identification of duck tissues [3]. During this process, biological material was investigated from KhO-1 (a breed of duck produced from Khaki Campbell males and Orpington fauve females), LsA (Pekin of English origin, produced from three English conservation flocks), P8 (produced from Danish conservation flocks), P9 (produced from French conservation flocks), P33 (produced from Polish conservation flocks, hybrids of Polish drakes, and Dutch ducks), Dworka duck (D11, a breed of duck obtained by crossing Cayuga males and Pekin females), Runner (a breed originating from Asia), and Rouen (a breed originating from France) ducks. For the Rouen breed, the PCR product was of low intensity and the limit of detection for this breed was in the higher range [2]. At the same time, the NCBI GenBank did not list the mitochondrial sequence of the Rouen duck, so it was impossible to check the primers for species specificity using the NCBI BLAST tool (http://www.ncbi.nlm.nih.gov/tools/primer-blast, accessed on 21 April 2021). Therefore, we attempted to determine a fragment of the mitochondrial rRNA gene sequence in the Rouen duck, which is most often used to identify this species.

Identifying duck DNA is very important because, during recent years, laboratories have received more and more requests to confirm the genetic origin of duck products (including Rouen duck products).

The Rouen breed was first introduced in France from the Muscovy duck. Currently, it occurs as the French and English types. The French version resembles a larger than average Mallard. By selective breeding, the British type developed to have a deep, long keel, boat-shaped profile. Rouen duck breeding is a rather limited, niche endeavor, but it is relevant in numerous local markets; representatives of the breed can satisfy the needs of poultry meat gourmets and ornamental poultry breeders. Rouen ducks are bred in many countries due to their meat’s excellent quality (production-type Rouen ducks); the meat is aromatic, juicy, delicate, and highly valued by connoisseurs. Due to their original ornamental appearance, Rouen ducks—formerly called “Giant Mallards”—can be found in many ornamental bird farms, and they are featured at ornamental poultry exhibitions (exhibition-type Rouen ducks). For these reasons, the species identification of Rouen ducks is an important scientific challenge.

The study aimed to determine the sequence of a fragment of the Rouen duck’s mtDNA sequence, to find universal primers for the determination of duck DNA regardless of breed, to find primers for distinguishing Rouen duck DNA compared to that of other breeds, and to check the developed primers.

## 2. Materials and Methods

### 2.1. Material

All of the procedures involved in collecting the research material complied with the Local Krakow Ethics Committee for Experiments with Animals’ guidelines as well as the Polish Act on the Protection of Animals Used for Scientific or Educational Purposes regulations of 15 January 2015 (which implements Directive 2010/63/EU of the European Parliament and the Council of 22 September 2010 on the protection of animals used for scientific purposes). The material used in this study was samples of meat and feathers from 16 (8 individuals of both French and English selection) Rouen ducks, duck DNA derived from feathers (KhO-1, LsA, P8, P9, P33, Dworka duck, and Runner duck), bird DNA derived from feathers (cockatiel, hoopoe, guinea fowl, peacock, turkey, hen, goose, and pigeon), mammalian DNA from meat (cattle, pig, sheep, and horse) and hair (dog and cat), and plant DNA (maize, different grains, and rice). Pekin duck meat was used as a positive control.

Feathers were derived from abandoned feathers in a birdcage at a pet store or in breeding places, and hairs were derived from abandoned hairs on a pet’s bed. Meat samples came from the slaughterhouse where the animals were slaughtered (according to Council Regulation (EC) No 1099/2009), and after carcass evaluation, the meat was intended for consumption.

### 2.2. DNA Isolation

DNA isolation from meat and plants was performed using the Ax Food kit (A&A Biotechnology, Gdańsk, Poland) according to the manufacturer’s procedure. DNA from hair and feather calami were extracted using the Sherlock kit (A&A Biotechnology, Gdańsk, Poland) according to the procedure for hair.

### 2.3. Sequencing a Part of Rouen Duck mtDNA

**Figure 1:**
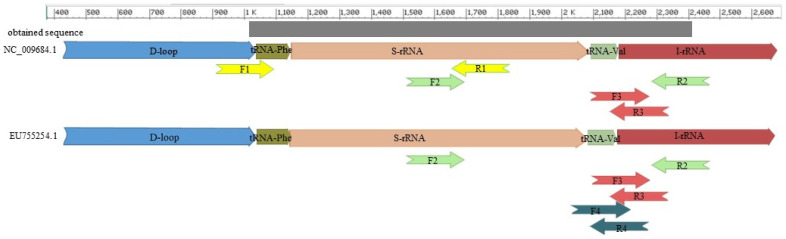
The place of the primers annealing to the mitochondrial genome of ducks and the sequences obtained.

The sequencing primers (the first and second pairs in Supplementary Table S1) were determined using the mitochondrial sequence of mallards (*Anas platyrhynchos*, NC_009684.1) and Muscovy ducks (*Cairina moschata,* EU755252) as a biological standards for modern ducks. In choosing the genome fragment for our analysis, we were guided by a previously determined mtDNA fragment that, for the Rouen duck, yielded a less intense PCR product [7]. The 966–2451 bp NC_009684.1 fragment chosen in the present study (Supplementary Table S1) surrounds the previously described 2025–2086 bp NC_009684.1 [7]. For the analyzed fragment of the mallard genome, primers were determined with the Primer3 program [8]. The designed primers enabled us to divide the identified sequence into two overlapping fragments. For the Muscovy duck, only the second primers allow one to obtain a DNA fragment analogous to mallard duck’s DNA. The sequences of the determined primers are shown in Table S1. The location of the primers annealing to the mitochondrial genomes of both breed is shown in Figure 1.

Then, the Rouen duck DNA obtained was separately amplified in the presence of primers F1/R1 and F2/R2 (Supplementary Table S1). The reaction mixture contained HotStarTaq DNA Polymerase, 1× PCR Buffer, 1× Q-Solution (Qiagen^®,^ Hilden, Germany), 1.33 mM MgCl2, and 0.67 μM of each primer. Amplification was performed in a Veriti Thermal Cycler (Thermofisher, Waltham, MA, USA) with the following cycling conditions; after an initial heat denaturation step at 96 °C for 5 min, 32 cycles were programmed as follows: 95 °C for 1 min, annealing temperature for 1 min, 72 °C for 1 min, and final extension at 72 °C for 15 min. The annealing temperatures were 52 °C and 56 °C for the first and second primer pairs, respectively. All polymerase chain reactions were performed with a negative PCR control (NTC) to detect potential contaminants in the reaction mixture and positive (DNA of Pekin duck) PCR control (PTC) to check the reaction results’ correctness. The products obtained were separated in 3% agarose gel.

Both strands of PCR product obtained were sequenced using a BigDye Terminator v3.1 Cycle Sequencing Kit in the presence of 5 ng of PCR product. The sequencing products were purified with a BigDye × Terminator kit (Thermo Fisher Scientific^®,^ Waltham, MA, USA) and subjected to capillary electrophoresis with an automated 3500 xl Genetic Analyzer sequencer (Applied Biosystems^®^, Foster City, CA, USA) using POP7 polymer (Applied Biosystems, Foster City, CA, USA), the rapid sequencing module.

The results were analyzed with Finch TV software v. 1.4.0 (GeospizaInc^®^; https://digitalworldbiology.com/FinchTV, accessed on 21 April 2021) and BioEdit v. 7.2 (Tom Hall; https://bioedit.software.informer.com, accessed on 21 April 2021). The obtained sequences were compared with the sequences deposited in GenBank using BLAST.

### 2.4. Checking the Developed Primers Specificity

To check the developed primers specificity, we performed a qPCR reaction using a 1× Quantum EvaGreen (Syngen BiotechSY550711,) for DNA obtained from the meat of three Rouen ducks. The concentration of each primer in the reaction mixture was 0.2 μM. For both primer pairs, we used a thermal program recommended by the Quantum EvaGreen manufacturer. The reactions were performed in three replicates for four consecutive dilutions of each matrix (factor 10). Ultimately, the reactions were performed for 10%, 1%, 0.1%, and 0.01% DNA dilutions, which corresponded to 5 ng, 0.5 ng, 0.05 ng, and 0.005 ng of DNA. For each regression curve, we determined a regression coefficient—slope (b), the value of CT (in the standard curve) where the regression line crosses the CT-axis cT-intercept, regression coefficient (R^2^), and amplification efficiency (EFF%). In addition, cross-reactions were performed for the DNA mix of birds (cockatiel, hoopoe, guinea fowl, peacock, turkey, hen, goose, and pigeon), mammals (cattle, pig, sheep, and horse), and plants (maize, different grains, and rice).

### 2.5. Development of Primers for Duck DNA Identification

Based on the mtDNA fragment determined by sequencing, two pairs of inner primers were designed. The first pair was complementary for both mallards (*Anasplatyrhynchos*) and Muscovy duck (*Cairina moschata)* (EU755252) (Supplementary Table S1, F3/R3, Figure 1). The second pair was complementary to the obtained sequence and *Cairina moschata* (Supplementary Table S1, F4/R4, Figure 1). The result of sequencing made it impossible to design primers only to sequence the DNA of Rouen duck. The primers showed no homology (or showed little similarity) to other farmed species (cattle, pig, sheep, chicken, turkey, goose, and guinea fowl). The primers were designed with Primer3 software [8].

PCR was performed under the same conditions as described previously. The annealing temperatures were 56 °C and 59 °C for the primers F3/R3 and F4/R4, respectively.

To check the primers, their range, biological specificity, and limit of detection (LOD) were determined. The species specificity of the primers was demonstrated using the DNA of ducks (Rouen duck; KhO-1; and a mix of Pekin duck, Dworka duck, and Runner duck), other birds (cockatiel, hoopoe, guinea fowl, peacock, turkey, hen, goose, and pigeon) mammals (cattle, pig, sheep, horse, dog, and cat), and plants (maize, various grains, and rice). The limit of detection was determined based on analyzing the DNA of several breeds of ducks at a dilution DNA of less than 0.5% (0.05 ng) and 0.05% (0.005 ng). Additionally, the specificity of the obtained PCR reaction products was checked in the sequencing reaction.

## 3. Results

### 3.1. Species Specificity of the Primers Used for Sequencing

Real-time PCR for the first and second primers yielded products for all Rouen ducks, and their mean CTs were very close to each other, differing by no more than 1.5 cycles. The regression curves generated from the consecutive dilutions, regardless of the matrix, had slopes of −3222 to −3525, intercepts of 25,031–26,710, regression coefficients of 0.980–1.000, and amplification efficiencies of 92,185–104,346 (Supplementary Table S2).

For the cross-reactions, the PCR product occurred several cycles later than the analyzed dilutions, which corresponded to the reaction specific for 0001% DNA (therefore, below the limit of detection) or did not occur at all (Supplementary Table S2).

### 3.2. PCR Reaction and Sequencing

A PCR product of approximately 1000 bp (Supplementary Figure S1) was obtained for both the first primer pair (wells 1–3 and 7) and the second primer pair (wells 4–6 and 8). The PCR products for meat (lanes 2 and 5), feathers (lanes 3 and 6), and positive PCR (lanes 7 and 8) control yielded an intense product in the form of a single band, whereas no reaction product was observed for the negative PCR control (lanes 1 and 4). Figure S1 presents the results for selected samples, only for one individual of the French selection.

### 3.3. Sequencing Part of the Rouen Duck mtDNA

**Figure 2:**
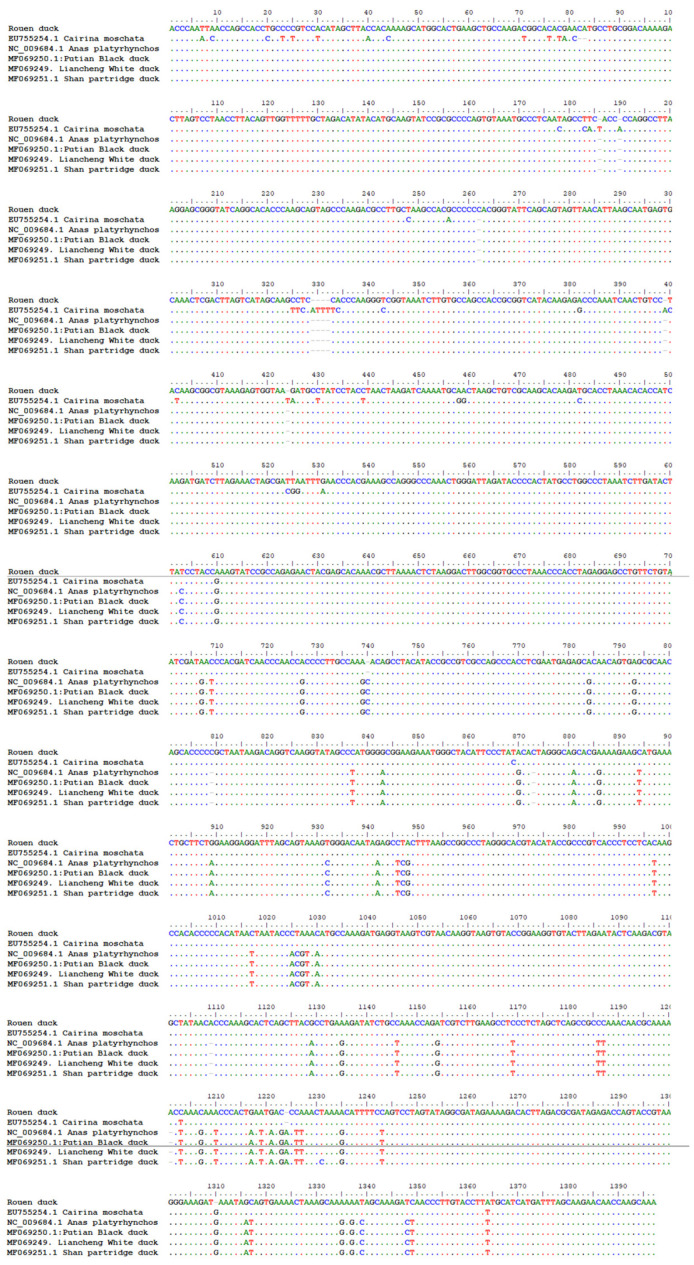
Multi-alignment sequences of duck DNA. The result of alignment sequences for the chosen duck breeds: *Anas platyrhynchos*, *Cairina moschata,* Putian black, Liancheng white, and Shan partridge.

The sequencing reaction yielded a consensus between the forward and reverse sequences and enabled a common consensus sequence, though it was shorter than expected, at 798–805 bp (depending on individual) for the first primer pair and 861–870 bp (depending on individual) for the second primer pair. Between the sequences samples, there were no differences in each selection (French and English) and between them. The sequences partially overlapped; therefore, ultimately, a sequence with a total length of 1386 bp was obtained. The obtained sequence (Figure 2) is shown in the next subsection.

The proposed sequence was 95% homologous to the mallard sequence (from 1025 to 2408 bp, *Anasplatyrhynchos*) and covered the d-loop, 12S rRNA, and 16S rRNA fragments. This sequence was also 98% homologous to Muscovy duck (from 1016 to 2407 bp, *Cairina moschata*), too and covered the d-loop, 12S rRNA, and 16S rRNA fragments. Part of this sequence was deposited in the NCBI GenBank under accession number MH078252.

### 3.4. Methods for the Identification of Duck DNA and Rouen Duck DNA

**Figure 3:**
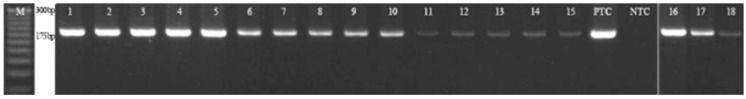
PCR result for the analyzed primers F3/R3. Results of the PCR reaction for the third primer pair. Lanes 1, 6, and 11—DNA of Rouen duck (French selection); lanes 16–18—DNA of Rouen duck (English selection); lanes 2, 7, and 12—DNA of KhO-1; lanes 3, 8, and 13—mix of Pekin duck DNA; lanes 4, 9, and 14—DNA of Dworka duck; and lanes 5, 10, and 15—DNA of Runner duck. Concentration of duck DNA: 100%—lanes 1–5; 0.5%—lanes 6–10; 0.05%—lanes 11–15, PTC—positive PCR control, NTC—negative PCR control, and M—the 25 bp step ladder.

**Figure 4:**
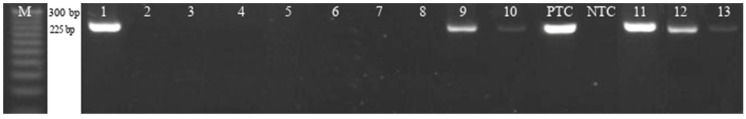
PCR result for the analyzed primers F4/R4. Results of the PCR reaction for the fourth primer pair. Lanes 1 and 11—100% DNA of Rouen duck; lane 2—DNA of KhO-1; lane 3—mix of Pekin duck DNA; lane 4—DNA of Dworka ducks; lane 5—DNA of Runner ducks; lane 6—DNA of other birds; lane 7—DNA of mammals; lane 8—DNA of plants; lanes 9 and 12—0.5% DNA of Rouen ducks; lanes 10 and 13—0.05% DNA of Rouen duck; lanes 1, 9, and 10—French selection; and lanes 11–13—English selection. PTC—positive PCR control, NTC—negative PCR control, and M— the 25 bp step ladder.

In the next phase of the investigation, we selected the third and fourth pairs of primers and used the third pair to detect duck DNA independently of breed of duck and the fourth pair specifically to detect DNA of only the Rouen duck. The PCR reactions using the third and fourth pairs of primers were carried out on DNA of ducks to verify the specificity of the primers. For the third primers, we generated specific fragments of 184–185 bp for all breeds of ducks (Figure 3), and for the fourth primers, we obtained a fragment specific only for Rouen duck (227 bp) (Figure 4). To detect possible cross-reactions, primers were tested in PCR with non-target species (other birds, mammals, and plants). In no case was a cross-reaction observed (Figure 4). Supplementary Figure S2 shows the results of the alignments of the PCR products of Rouen and Pekin duck obtained by using primers 3 and 4 as well as the sequences of *Anas platyrhynchos* and *Cairina moschata*. The homology of the PCR reaction products of Rouen duck and the third pair of primers is 100% similarity to the part of the sequence of Rouen duck DNA identified by us and 99% similarity to the mtDNA of *Cairina moshata*, while the analogous reaction for the Pekin duck yielded a sequence 100% homologous with the mtDNA *Anas platyrhynchos*. Between the obtained sequences of Rouen and Pekin duck, we observed differences resulting from variations in their genome. Moreover, the PCR product of the fourth pair of primers and the DNA of the Rouen duck was nearly 100% identical to the sequence of the mitochondrial DNA of *Cairina moschata*.

## 4. Discussion

Mitochondrial DNA was used in this analysis due to the large number of copies in every cell and the sequence stability within species. However, genome research in ducks indicates that single-nucleotide substitutions occur between individual breeds within a species. This phenomenon has been observed in duck populations from Europe, Asia, and America, and it concerns diverse mtDNA fragments. Susanti et al. described the pattern within the gene encoding COX I [9]. Based on genomic studies of seven duck breeds living in Indonesia, the authors identified nine variable sites and five haplotypes in the above gene [9]. The d-loop is another genome fragment in which several nucleotide substitutions have been reported. Based on D-loop fragment research in ducks from Bangladesh and Korea, 16 nucleotide substitutions have been detected, making the identification of 28 haplotypes possible [10]. A similar study with Magelang, Tegal, Mojosari, Bali, and Alabio ducks from Indonesia confirmed the substitutions of the D-loop fragment [11].

The substitution observed is a natural phenomenon. It allows for the determination of an animal’s breed identity, its occurrence at a given latitude, or the origin of various breeds of ducks—for example, Eurasian and American [12]—with a high degree of probability. Eurasian and American ducks have a completely different genetic structure, and the European and Asian populations share only a single haplotype [12]. In turn, ducks on the same continent or in the same country are characterized by low polymorphism compared to the polymorphism between individuals coming from distant locations [12,13]. The present experiment yielded similar observations. The sequenced Rouen duck fragments are 97% homologous with the appropriate sequence of the Muscovy duck (*Cairina moschata*, EU755254), while the same sequences are 95% similar to sequences of Asian breeds—for example, the Putian black, Liancheng white, Shan partridge, Xilin duck, Lin Wu, Longsheng duck, and Rongshui duck. The differences in the form of single substitutions and deletions are shown in Figure 2. In the first part, the sequence is more similar to *Anasplatyrhynchos* and the Chinese breeds, while in the following part, it is closer to *Cairinamoschata*. A comparison with the sequences of Khaki Campbell duck, Cayuga duck, and Runner duck was impossible because NCBI includes no analogous sequences of these breeds.

The sequence that this study detected could contribute to improving the methods of species identification for duck mtDNA. Appropriate primers can be designed to distinguish between the Rouen duck and other ducks, or a method can be developed to identify Rouen ducks in the same way as other breeds. The designated primers (1 and 2) are species-specific: with the CT-intercept for all of the analyzed samples being in the 25–26 range. The presence of the reaction product for nonspecific DNA species was irrelevant as it corresponded to an often-lower concentration than the assumed limit of detection.

All of the pairs of primers presented in this study are 100% identical to the sequences of ducks used in the study; therefore, regardless of breed, an intense PCR product ensuring high reaction sensitivity was obtained in all reactions. By contrast, earlier primers employed to determine duck DNA [14] were 83% (forward) and 95% (reverse) homologous to the sequences limiting the amplified fragment. It can be assumed that such a large difference translated into a noticeable effect on the reduced reaction yield of duck DNA [6].

As no mtDNA sequence for the Rouen duck was deposited in the NCBI database, we attempted to identify such a sequence.

The sequence reported, with a total length of 1386 bp, is found in the d-loop, 12S rRNA, and 16S rRNA gene loci, and it differs by around 7% from the sequences of the other domestic duck species under study (*Anasplatyrhynchos*). The sequence detected contributes to improving the methods for species identification of duck mtDNA.

The primers developed on this basis aid in detecting the DNA of various breeds of ducks, or vice versa, specifically Rouen duck alone. The sensitivity of the primers is very high regardless of duck breed and the differences in their sequences. The tests developed are suitable for analyzing mixtures of biological material for the content of duck DNA. The primers designed and described in this paper were proven to be highly sensitive, with a very low detection limit (1%). The first one, in contradistinction to earlier known methods of the identification of duck DNA [2,14], allows for detecting the DNA of ducks independent of their breed. The limit of detection is the same for the Rouen duck as for other ducks. An important aspect of the present study is that the literature reports no methods to distinguish various breeds of ducks based on one reaction. The fourth pair of primers presented by us enable distinguishing Rouen and Muscovy ducks from other breeds.

All of the proposed primers are suitable for identifying duck DNA (from both meat and feathers) due to their species specificity and low limit of detection, but due to PCR product length, the tests using the third and fourth primers have the most practical applications. An amplicon length of less than 200 bp guarantees that a PCR product is obtained regardless of the template and its possible degradation.

## 5. Conclusions

The mtDNA sequence identified for the Rouen duck can aid in the design of methods for detecting duck DNA regardless of breed as well as methods that distinguish purebred Rouen ducks from other breeds of this species. The primers proposed have great potential as a molecular tool that can be used for the routine detection of duck DNA. The third pair of primers proposed can detect duck tissue in the range of 5–100%, regardless of the differences in sequences between the breeds. The fourth pair of primers proposed can distinguish the DNA of Rouen and Muscovy ducks from that of Chinese ducks. The limit of detection is 0.5%, which corresponds to 25 pg of the template DNA.

## Data Availability

The data presented in this study are available in the NCBI GenBank under accession number MH078252.

## Supplementary Materials

The following are available online at https://www.mdpi.com/article/10.3390/genes12060857/s1, Figure S1. PCR result for analysed primers F1/R1, F2/R2. Figure S2. Multi alignment obtained sequences by using third and fourth primers pair. Table S1. Sequences of primers used to determine part of mtDNA for the Rouen duck. Table S2. Parameters of the regression curves.
