# Supplementary material for: Mitochondrial Markers for the Detection of Duck Breeds Using Polymerase Chain Reaction
Source: Genes (Basel). 2021 Jun 3;12(6):857. doi: 10.3390/genes12060857 (PMC8228690; doi:10.3390/genes12060857)
Supplement: Supplementary file 1 [file genes-12-00857-s001.zip › genes-1211922-supplementary.pdf]

Table 1. Sequences of primers used to determine part of mtDNA for the Rouen duck.

Primer Pair	Mtdna Region		Amplicon Length	Annealing Temp
F1: TCCAGCAC-TAAAGACGATCC R1: CCCATTTCTTCCGTCCCATA	NC_009684.1	D-loop <966..1049<br>tRNA 1050..1119<br>rRNA 1120..1870>	905 bp	52 °C
	EU755254.1	No PCR product	-	56 °C
F2:AGATGCAC-CTAAACACACCA R2:TAAGTAGCTCGCTTGGGTTT	NC_009684.1	rRNA<1494..2104<br>tRNA 2105..2175<br>rRNA 2176..2451>	958 bp	56 °C
	EU755254.1	rRNA<1491..2102<br>tRNA 2103..2174<br>rRNA 2175..2449>	959 bp	59 °C
F3:ACCCAAAGCAC-TCAGCTT R3:TACTGGTCTC-TATCGCGTCT	NC_009684.1	tRNA<2122..2173<br>rRNA 2174..2305	184 bp	
	EU755254.1	tRNA<2120..2174<br>rRNA 2175..2304	185 bp	
F4:CACCCCCACATAACTAATACCC R4:AGTTT-GGGTCATTCAG-TGGG	NC_009684.1	No PCR product	-	
	EU755254.1	rRNA<2018..2102<br>tRNA 2103..2174<br>rRNA2175..2241>	224 bp	
	Obtained sequence	<487–713>	227 bp	

Table 2. Parameters of the regression curves.

Primers 1									
Sample	Coefficients of simple regression					Quantity of DNA mixture of			
	slope	C_T -Inter-cept	R^2	Eff [%]	LOD	birds	mammals	plants	water
1	-3.337	25.031	0.983	100.694	0.025	0.00	0.00	0.00	0.00
2	-3.224	25.078	0.980	104.265	0.028	0.00	0.00	0.00	0.00
3	-3.222	26.154	0.998	104.346	0.024	0.00	0.00	0.00	0.00
Primers 2									
Sample	Coefficients of Simple Regression					Quantity of DNA mixture of			
	slope	C_T -Inter-cept	R^2	Eff [%]	LOD	birds	mammals	plants	water
1	-3.273	26.406	0.988	102.066	0.024	0.00	0.00	0.00	0.00
2	-3.525	26.119	0.980	92.185	0.024	0.00	0.00	0.00	0.00
3	-3.367	26.710	1.000	98.153	0.005	0.00	0.00	0.00	0.00

Slope-regression coefficient: In the standard curve, the value of CT where the regression line crosses the CT-axis – CT-intercept, Regression coefficient – R2, amplification efficiency – EFF, limit of detection – LOD.Quantity of DNA of plants and other groups of animals determined by the standard curve (cross-reactions) - Quantity of DNA mixture.

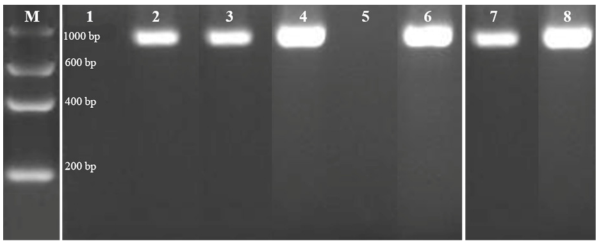

Figure S1. PCR result for analysed primers F1/R1, F2/R2. Results of PCR reaction of first (wells 1–3, 7) and second primer pair (wells 4–6, 8). Lanes 1 and 5 – negative PCR control, lanes 2 and 4 – products for English selection Rouen's meat, lanes 3 and 6 – products for English selection Rouen's feathers, lanes 7 and 8 – positive PCR control. M-200 -1000 bp step ladder.

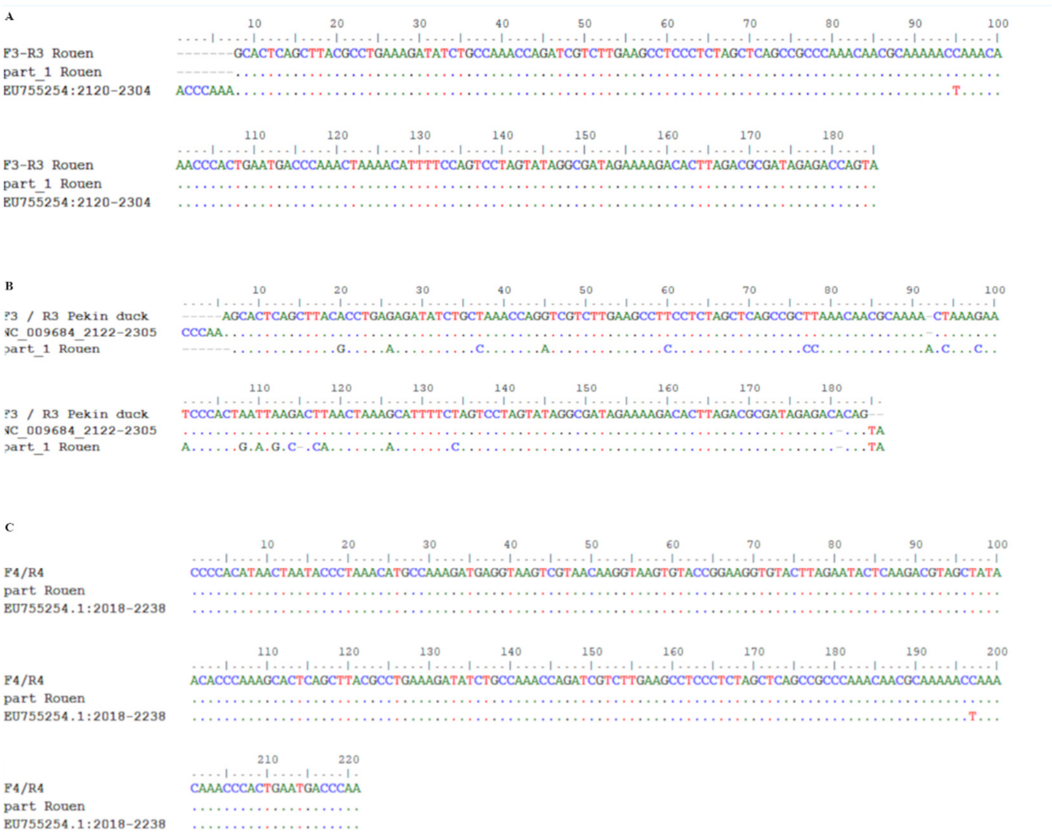

Figure S2. Multi alignment obtained sequences by using third and fourth primers pair. Result of alignment: **A**: sequences flanked by using third pair of primers; obtained sequences of Rouen duck (F3-R3 Rouen), part of identified by us sequence of Rouen duck (part_1 Rouen), *Cairina moschata* (EU755254:2120-2304); **B**: sequences flanked by using third pair of primers; obtained sequences of Pekin duck (F3/R3 Pekin duck), part of identified by us sequence of Rouen duck (part_1 Rouen), *Anas platyrhynchos* (NC_009684_2122-2305); **C**: sequences flanked by using forth pair of primers; obtained sequences of Rouen duck (F4-R4), part of identified by us sequence of Rouen duck (part Rouen), *Cairina moschata* (EU755254:2018-2238).
